# *Strongyloides stercoralis* and hookworm co-infection: spatial distribution and determinants in Preah Vihear Province, Cambodia

**DOI:** 10.1186/s13071-017-2604-8

**Published:** 2018-01-12

**Authors:** Armelle Forrer, Virak Khieu, Fabian Schär, Penelope Vounatsou, Frédérique Chammartin, Hanspeter Marti, Sinuon Muth, Peter Odermatt

**Affiliations:** 1 Swiss Tropical and Public Health Institute, Basel, Switzerland; 2 University of Basel, Basel, Switzerland; 3 National Centre for Parasitology, Entomology and Malaria Control, Ministry of Health, Phnom Penh, Cambodia

**Keywords:** Strongyloidiasis, Hookworm, Co-infection, Spatial, Bayesian, Helminths, Control, Risk profiling, Cambodia

## Abstract

**Background:**

*Strongyloides stercoralis* and hookworm are two soil-transmitted helminths (STH) that are highly prevalent in Cambodia. *Strongyloides stercoralis* causes long-lasting infections and significant morbidity but is largely neglected, while hookworm causes the highest public health burden among STH. The two parasites have the same infection route, i.e. skin penetration. The extent of co-distribution, which could result in potential high co-morbidities, is unknown in highly endemic settings like Cambodia. The aim of this study was to predict the spatial distribution of *S. stercoralis*-hookworm co-infection risk and to investigate determinants of co-infection in Preah Vihear Province, North Cambodia.

**Methods:**

A cross-sectional survey was conducted in 2010 in 60 villages of Preah Vihear Province. Diagnosis was performed on two stool samples, using combined Baermann technique and Koga agar culture plate for *S. stercoralis* and Kato-Katz technique for hookworm. Bayesian multinomial geostatistical models were used to assess demographic, socioeconomic, and behavioural determinants of *S. stercoralis*-hookworm co-infection and to predict co-infection risk at non-surveyed locations.

**Results:**

Of the 2576 participants included in the study, 48.6% and 49.0% were infected with *S. stercoralis* and hookworm, respectively; 43.8% of the cases were co-infections. Females, preschool aged children, adults aged 19–49 years, and participants who reported regularly defecating in toilets, systematically boiling drinking water and having been treated with anthelmintic drugs had lower odds of co-infection. While *S. stercoralis* infection risk did not appear to be spatially structured, hookworm mono-infection and co-infection exhibited spatial correlation at about 20 km. Co-infection risk was positively associated with longer walking distances to a health centre and exhibited a small clustering tendency. The association was only partly explained by climatic variables, suggesting a role for underlying factors, such as living conditions and remoteness.

**Conclusions:**

Both parasites were ubiquitous in the province, with co-infections accounting for almost half of all cases. The high prevalence of *S. stercoralis* calls for control measures. Despite several years of school-based de-worming programmes, hookworm infection levels remain high. Mebendazole efficacy, as well as coverage of and compliance to STH control programmes should be investigated.

**Electronic supplementary material:**

The online version of this article (10.1186/s13071-017-2604-8) contains supplementary material, which is available to authorized users.

## Background

*Strongyloides stercoralis* and hookworms are parasitic intestinal nematodes that belong to the group of soil-transmitted helminths (STH). For both parasites, infection occurs when larvae living in faecally-polluted soil penetrate intact skin. STH mostly affect the poorest, locking them into poverty through a cycle of gastro-intestinal symptoms, malnutrition and long-term impact on fitness and productivity [1–3]. With overlapping geographical distributions, STH are mostly prevalent in rural areas with poor sanitation conditions and a warm and humid climate that favour larvae survival in the environment [2, 4, 5].

Hookworm infection is of major public health importance in low and middle-income countries, with 439 million cases reported worldwide in 2010 [6]. Hookworm causes the highest burden among STH, with detrimental effects on children’s physical and cognitive development and agricultural workers’ productivity. It also gives rise to hookworm disease, which occurs in cases of high worm load and causes iron-deficiency anaemia that affects infant and maternal mortality and leads to low birth weights [2, 3, 7, 8].

As for *S. stercoralis*, it is one of the most neglected of the neglected tropical diseases (NTDs), mostly because its larvae, present in human stool, are not detected by the diagnostic coprological techniques used in endemic countries to screen for helminth eggs [5, 9–11]. However, the parasite has recently gained some attention from the scientific and global health community and recent studies in Cambodia found high prevalence rates of up to 45% [12–14]. Not only is this parasite very common, but it also causes significant dermatological and gastro-intestinal morbidity and is associated with chronic malnutrition in children [15, 16]. Moreover, its ability to replicate within its host leads to long-lasting infections and potentially fatal dissemination of the parasite [17–19].

The backbone of the WHO strategy to control STH is preventive chemotherapy (PC), i.e. regular treatment to prevent high-intensity infections and associated morbidity administered to entire populations or at-risk groups. In the case of hookworm, children and women of childbearing age are particular targets for treatment [20, 21]. However, continued exposure to contaminated environments due to unhygienic behaviour leads quickly to re-infection [22–24] and re-treatment is required.

To date, *S. stercoralis* is not included in the WHO recommended preventive chemotherapy control strategy used against STH, in which anti-helminthic treatment is the main pillar. The drug of choice against *S. stercoralis*, ivermectin, is safe, well tolerated and highly efficacious [25–27]. It was recently found that a single oral dose (200 μg/kg body weight (BW)) of ivermectin achieved a high cure rate and resulted in re-infection rates below 15%, one year after treatment in a highly endemic setting in Cambodia [24–26].

Both *S. stercoralis* and hookworm are highly prevalent in Preah Vihear Province [28, 29]. Considering the need to integrate *S. stercoralis* control into existing STH programmes, it is important to know the extent to which the distributions of the two parasites overlap geographically and across age groups. Geostatistical models using survey parasitological data combined with remote sensing (RS) environmental data provide unique tools for estimating parasite distribution over small or large areas, thereby providing a rapid and cost-effective means of identifying the areas of greatest need and guiding control efforts [30–32].

The aim of the present work was to assess the geographical distribution and explore the underlying factors of *S. stercoralis*-hookworm co-infection risk in the rural Province of Preah Vihear, northern Cambodia.

## Methods

### Study setting and design

The study was conducted among the general population of Preah Vihear Province, located in northern Cambodia and bordering Thailand. With a population of 171,139 in 2008 and a total area of 16,132 km^2^, the province stretches from 13°06′ to 14°44’N, 104°37′ to 106°91′E [33]. The poverty incidence, defined as the proportion of individuals living in households with an average per capita expenditure below the poverty line of 6347 riels (1.55 USD), was 33.5% in 2009 [34]. It has a monsoon type climate, with a rainy season occurring from May to October. *Strongyloides stercoralis* and hookworms, as well as protozoa, are highly endemic in this region [12]*.*

Sixty of the 184 villages in Preah Vihear province were selected for a large-scale, cross-sectional study carried out from February to June 2010. Six of seven districts were included; the district of Chaeb was excluded because the distance between its villages and the study laboratories was too large to ensure sufficiently fast transfer of samples to preserve their integrity. In each village, 15 households were randomly selected from the list of all households and all household members aged one and over were eligible for inclusion in the study.

### Parasitological data and case definition

Two stool samples were collected on consecutive days from each participant. *Strongyloides stercoralis* was diagnosed using both Koga agar plate (KAP) culture and the Baermann technique performed on each sample [35, 36]. A detailed description of this laboratory procedure is given elsewhere [12]. Hookworm was diagnosed using Kato-Katz thick smears, KAP culture and the Baermann technique on each sample. *Strongyloides stercoralis* larvae and hookworm eggs were identified through microscope examination and based on morphology. For quality control, technicians were specifically trained to differentiate *S. stercoralis* and hookworm larvae. In addition, they were rigorously supervised by a qualified microscopist from the Swiss Tropical and Public Health Institute (Swiss TPH), Basel, Switzerland. Any unclear diagnosis was immediately discussed with both the qualified microscopist and the study supervisor.

In this analysis, infection status was defined as follows. For *S. stercoralis*, a participant was considered positive if at least one larva was found in any of the four samples (KAP and Baermann technique on two samples) or negative if no larva was detected in the four samples. Participants with only negative results but with fewer than four analysed samples were not included in the analysis. For hookworm, a participant was considered positive if at least one egg was found in any of the six samples (Kato-Katz, KAP, and Baermann technique on two samples), and negative if both Kato-Katz slides were negative. Negative participants with one missing Kato-Katz examination were not included in the analysis. All reported results in this paper use the definitions described above.

### Demographic, socioeconomic, knowledge and hygiene practices data

Data on demographic factors (age, sex, main occupation, level of education), hygiene practices (hand washing, shoe wearing, regular place of defecation), and worm-related knowledge (sources of infection, health problems caused by worms) were collected with an individual questionnaire. Heads of households were administered an additional questionnaire to collect information on household size, water and sanitation conditions, house material and household asset ownership. Age was categorized into four classes, as follows: (i) < 6 years; (ii) 6–18 years; (iii) 19–49 years; and (iv) ≥ 50 years. About 17% (432/2576) of the participants reported their occupation as “other”, most of whom declared being at home (361/432), while the remaining occupations varied from teachers, nurses and military, to village chiefs and construction workers. Original categories of variables with frequencies under 5% were grouped with similar categories.

An asset-based socioeconomic index was built using house construction material, ownership of household assets and multiple correspondence analysis (MCA), a data reduction technique that was developed for categorical data [37, 38]. Households were classified into one of three wealth classes, from the least poor to the poorest.

### Environmental data

Environmental parameters were extracted from freely available remote sensing (RS) sources. Day and night land surface temperature (LST), land use/land cover (LULC), and enhanced vegetation index (EVI) were extracted at 1 × 1 km resolution from Moderate Resolution Imaging Spectroradiometer (MODIS) Land Processes Distributed Active Archive Center (LP DAAC), U.S. Geological Survey (USGS) Earth Resources Observation and Science (EROS) Center (http://lpdaac.usgs.gov). EVI was used instead of the normalized difference vegetation index (NDVI), as it is more sensitive to differences in heavily vegetated areas and, thus, more appropriate for Southeast Asia (see http://earthobservatory.nasa.gov/Features/MeasuringVegetation/measuring_vegetation_4.php). Rainfall estimates (RFE) at 0.1 degree (about 10 × 11 km) resolution were obtained from the National Oceanic and Atmospheric Administration’s (NOAA) Climate Prediction Center (CPC) Famine Early Warning System (FEWS) Rainfall Estimates South Asia, version 2.0 (http://www.cpc.ncep.noaa.gov/products/international/). Digital elevation data at a resolution of 90 × 90 m were retrieved from the NASA Shuttle Radar Topographic Mission’s (SRTM) Consortium for Spatial Information of the Consultative Group for International Agricultural Research (CGIAR-CSI) database. Soil type data, including bulk density, soil organic carbon content and pH, at a spatial resolution of 9 × 9 km, were extracted from the International Soil Reference and Information Center’s (ISRIC) World Inventory Soil Emission Potentials (WISE), version 1.0 (http://www.isric.org). District information (i.e. district name) was downloaded from the Global Administrative Areas website (http://www.gadm.org). The 18 land cover type 1 classes (IGBP) were merged into five categories, according to similarity and respective frequencies. Yearly means, as well as minima and maxima of EVI, monthly LST and RFE were calculated for May 2009 to April 2010.

### Statistical analysis

ArcGIS version 10.0 (ESRI; Redlands, CA, USA) was used for environmental data processing, geo-referencing and map drawing. Environmental data was linked to parasitological and questionnaire data according to location. Data management was performed in STATA version 13.0 (StataCorp LP; College Station, USA).

Bayesian mixed multinomial models, i.e. with a random effect accounting for village-level clustering, were used to jointly model the risks of mono-infection with *S. stercoralis*, mono-infection with hookworm, and *S. stercoralis*-hookworm co-infection. Such a model yields relative risk ratios for categorical outcomes (here, the two mono-infection and the co-infection risks) compared to a baseline outcome (in the present case, “no infection”). Two types of Bayesian mixed multinomial models were developed. First, a model using demographic, socioeconomic and behavioural data was developed to assess determinants of *S. stercoralis* and hookworm mono- and co-infection. Second, a model including only environmental covariates aimed to predict *S. stercoralis*-hookworm mono- and co-infection risks at non-surveyed locations in Preah Vihear province.

Variable selection for both types of models was done in STATA using simple multinomial models, based on 15% significance level as assessed by the likelihood ratio test (LRT). In case of correlation, the variable resulting in the model with the smallest Akaike’s information criterion (AIC) was selected. Selected continuous covariates were standardized. We checked whether sex was an effect modifier of any other variable in the model.

A mixed logistic regression model for *S. stercoralis* infection was used to estimate the odds ratio for co-infection with hookworm; this was done in STATA, while adjusting for all the variables present in the determinant analysis. The unadjusted odds ratio was produced using a mixed bivariate logistic regression.

Bayesian multivariate mixed multinomial models were fitted using OpenBUGS version 3.2.3 (Imperial College & Medical Research Council, London, UK) [39]. Models without or with environmental covariates were run with either a spatial (geostatistical) random effect, using the OpenBUGS “spatial.unipred” function, or with an exchangeable random effect. To explore the clustering tendency of *S. stercoralis* and hookworm mono- and co-infection risks, a Bayesian spatial multinomial model was run in the absence of covariates. Spatial models assumed a stationary isotropic process, with village-specific random effects following a normal distribution with mean zero and a variance-covariance matrix being an exponential function of the distance between pairs of locations. Vague prior distributions were chosen for all other parameters. Markov Chain Monte Carlo (MCMC) simulation was used to estimate model parameters [40]. Convergence was assessed by examining the ergodic averages of selected parameters. Further information about models specification is available in Additional file 1. For all models, a burn-in of 5000 was followed by 30,000 iterations, after which convergence was reached. Results were withdrawn for the last 10,000 iterations of each chain, with a thinning of 10.

For model validation, 48 randomly selected villages were used for fitting and the 12 remaining villages were used as test locations. Model predictive ability was assessed with the Mean Squared Error (MSE), which was obtained for test locations by squaring the average of absolute differences between predicted and observed prevalence rates.

Based on environmental factors only and using Bayesian kriging, predictions of *S. stercoralis* mono-infection, hookworm mono-infection and *S. stercoralis*-hookworm co-infection risks at non-surveyed locations were made at 16,532 pixels of a 1 × 1 km resolution [41].

## Results

### Study population and size

Of the 3560 participants with available questionnaire data, 221 did not provide any stool sample. The case definition used in the present study resulted in the further exclusion of 662 *S. stercoralis* negative participants with fewer than four available diagnostic tests (KAP and Baermann on two samples) and 101 hookworm negative participants with fewer than two available diagnostic examinations (Kato-Katz on 2 samples), resulting in a sample of 2576 participants. While this sample was used for the predictive model, the final sample for the determinant analysis consisted in 2502 participants (70.3%) with complete questionnaire data and covering in 769 households and 60 villages. The characteristics of those 2502 participants are presented in Table 1.

**Table 1:** Characteristics of participants included in the analysis

Variable	Category	*n* (%)
Sex	Male	1095 (43.8)
	Female	1407 (56.2)
Age (years)	< 6	193 (7.7)
	6–18	992 (39.7)
	19–49	1015 (40.6)
	≥ 50	302 (12.1)
Occupation	Rice farmer	1216 (48.6)
	School	817 (32.7)
	At home other	469 (18.7)
Level of education	No school	821 (32.8)
	Primary school	1450 (58.0)
	Secondary school or higher	231 (9.2)
Socioeconomic status	Least poor	918 (36.7)
	Poor	815(32.6)
	Poorest	769 (30.7)
Ever treated for worms	Yes	1754 (70.1)
	No or don’t know	748 (29.9)
Reported regular defecation place	Forest	908 (36.3)
	Toilet	293 (11.7)
	Rice field or water	302 (12.1)
	Behind the house	999 (39.3)
Wearing shoes, frequency	Often or always	2139 (85.5)
	Sometimes or never	363 (14.5)
Washing hands before eating	Yes	2304 (92.1)
	No	198 (7.9)
Washing hands after defecating	Yes	1834 (73.3)
	No	668 (26.7)
Using soap or ashes when washing hands	Yes	768 (30.7)
	No	1734 (69.3)
Boiling drinking water	Never	1841 (72.3)
	At dry or wet season, but not both	191 (7.5)
	Yes, both seasons	514 (20.2)
Do you know anything about worms?	No	2016 (80.6)
	Yes	486 (19.4)
Ever used health facility	Yes	1935 (77.3)
	No	567 (22.7)
Distance to health facility (minutes)	Close (1 to 20 min)	701 (28.0)
	Less close (21 to 30 min)	729 (29.1)
	Least close (≥ 31 min)	502 (20.1)
	Not applicable	570 (22.8)
Toilet at home	No	2216 (88.6)
	Yes	286 (11.4)
Own dog	No	873 (34.9)
	Yes	1629 (65.1)
Own farm animals	No	201 (8.0)
	Yes	2301 (92.0)
Main water source for general use	Open water body^a^, rain	178 (7.1)
	Well	1525 (61.0)
Open water: pond canal river lake dam	Well pump	799 (31.9)
District	Tbang Mean Chey	559 (22.3)
	Rovieng	626 (25.0)
	Chey Saen	354 (14.2)
	Choam Khsant	402 (16.1)
	Sangtom Thmei	248 (9.9)
	Kulean	313 (12.5)
Land use/land cover	Savanna	769 (30.8)
	Forests	300 (12.0)
	Grassland	191 (7.6)
	Cropland and crop-natural vegetation mosaic	1242 (49.6)
Soil organic carbon (g/kg)	5.00–9.99	1190 (47.6)
	10.00–19.99	1312 (52.4)
	median (IQR)	
LST day year minimum (°K)	299.4 (2.1)	
LST night year mean (°K)	296.2 (0.7)	
Rainfall year maximum (mm/day)	16.7 (1.1)	

Data were obtained from a cross-sectional survey conducted in 2010 in 60 villages of Preah Vihear Province, North Cambodia, among 2502 participants aged over 1 year

^a^Open water body: pond, canal, river, lake, dam

*Abbreviations: IQR* interquartile range, *LST* land surface temperature

The proportion of males and females, and those who had ever been treated for worms were similar in the groups of excluded and included participants. Children under the age of six were less represented in the analysed sample (7.7%) than in the group of excluded participants, due to missing diagnostic results (24.6%). This was also reflected in the variable “occupation”, for which participants spending their time “at home” were also less represented. This group of participants consisted mostly of children, as adults were evenly distributed in the two groups. Participants declaring regularly defecating in toilets or behind their house were less represented in the included group, as well as those declaring wearing shoes “sometimes or never”. All those variables were therefore adjusted for in the multivariate model, with the exception of occupation, which was collinear with age and thus removed from the model (see below).

### Strongyloides stercoralis and hookworm prevalence

About two in three (1749/2576) participants were infected with *S. stercoralis* (1252 cases) or/and hookworms (1263 cases). The overall prevalence of *S. stercoralis* was 48.6% [95% confidence interval (CI): 46.7–50.6%], with 61.2% (95% CI: 58.4–63.9%) of *S. stercoralis* cases being co-infected with hookworms. The overall prevalence of hookworm was 49.0% (95% CI: 47.1–51.0%), with 60.6% (95% CI: 57.9–63.4%) being co-infected with *S. stercoralis*. Of the 1089 hookworm cases for which egg counts were available (i.e. 86.2% of hookworm cases with at least one positive Kato-Katz slide), most (1044/1089, 95.9%) were of light intensity according to the WHO classification [1–1999 eggs/g (epg)], whereas the remaining infections were either of moderate (2.0%) or heavy (2.1%) intensity. Individuals infected with *S. stercoralis* had double the odds of being infected with hookworm [unadjusted odds ratio (OR): 2.49 (95% CI: 2.10–2.97), adjusted OR: 2.21 (95% CI: 1.84–2.66)].

### Determinants of *S. stercoralis* and hookworm mono- and co-infection risks

The results of bivariate multinomial regressions are available in Additional file 2: Table S1. Occupation and age were collinear, and occupation was removed from the model since it was not a confounder of any other variable and its removal yielded a lower AIC in the multivariate model (AIC with occupation: 6314.8, AIC without occupation: 6311.2). No interaction was found.

Table 2 presents the results of the multivariate Bayesian mixed multinomial regression, accounting for village heterogeneity and jointly assessing determinants of each parasite mono- infection or co-infection as compared to non-infected participants. Females had lower odds of any infection type. The risk of being infected with *S. stercoralis* peaked among participants over 50 years old, the odds of hookworm mono-infection was lower for preschool-aged (age < 6 years) children, and co-infection risk was lower among preschool-aged children and adults between 19 and 49 years old. The poorest individuals had higher odds of either mono-infection or co-infection. Toilet use was a protective factor against hookworm mono-infection or co-infection, while co-infection risk was higher among participants living at larger distances from a health centre.

**Table 2:** Determinants of hookworm and *S. stercoralis* mono- and co-infection. RRR in bold were significant at 5% level. Data were obtained from a cross-sectional survey conducted in 2010 in 60 villages of Preah Vihear Province, North Cambodia, among 2502 participants aged 1 year and older

Variable	Category	*S. stercoralis* mono-infection		Hookworm mono-infection		Co-infection	
		RRR	95% BCI	RRR	95% BCI	RRR	95% BCI
Sex	Female	1.00		1.00		1.00	
	Male	**2.01**	1.56–2.54	**1.47**	1.15–1.88	**2.06**	1.65–2.62
Age (years)	6–18	1.00		1.00		1.00	
	< 6	0.69	0.40–1.22	**0.29**	0.15–0.52	**0.25**	0.15–0.40
	19–49	1.18	0.88–1.56	1.06	0.79–1.41	**0.74**	0.56–0.97
	≥ 50	**1.59**	1.06–2.37	0.87	0.57–1.33	0.86	0.57–1.27
Socioeconomic status	Least poor	1.00		1.00		1.00	
	Poor	1.1	0.82–1.49	0.79	0.58–1.08	1.13	0.84–1.51
	Poorest	**1.56**	1.13–2.17	**1.49**	1.07–2.05	**1.67**	1.22–2.33
Own dog	No	1.00		1.00		1.00	
	Yes	1.21	0.92–1.60	0.99	0.76–1.29	0.89	0.68–1.16
Reported regular defecation place	Forest	1.00		1.00		1.00	
	Toilet	0.65	0.41–1.01	**0.51**	0.31–0.83	**0.43**	0.25–0.71
	Rice field of water	0.84	0.54–1.31	0.72	0.44–1.16	0.92	0.62–1.37
	Behind the house	0.9	0.66–1.27	1.1	0.79–1.52	1.17	0.84–1.61
Boiling drinking water	Never	1.00		1.00		1.00	
	Yes during dry or wet season but not both	1.13	0.66–2.02	**1.9**	1.18–3.12	1.29	0.77–2.15
	Yes both dry and wet season	1.14	0.81–1.63	0.78	0.54–1.13	**0.67**	0.47–0.95
Wearing shoes, frequency	Often or always	1.00		1.00		1.00	
	Sometimes or never	**0.47**	0.29–0.76	0.71	0.46–1.09	0.88	0.61–1.30
Do you know anything about worms?	No	1.00		1.00		1.00	
	Yes	1.29	0.92–1.85	1.1	0.78–1.58	**1.48**	1.06–2.07
Distance to health facility (minutes)	Close (1–20)	1.00		1.00		1.00	
	Less close (21–30)	1.06	0.73–1.55	1.18	0.77–1.71	**1.53**	1.07–2.28
	Least close (≥ 31)	1.1	0.70–1.80	1.3	0.78–2.12	**1.7**	1.09–2.73
	Not applicable^b^	0.9	0.62–1.33	1.13	0.76–1.67	1.32	0.88–1.96
Ever treated for worms	No or don’t know	1.00		1.00		1.00	
	Yes	0.79	0.59–1.06	**0.58**	0.43–0.77	**0.43**	0.32–0.57
District	Tbaeng Mean Chey	1.00		1.00		1.00	
	Rovieng	0.78	0.40–1.27	**0.29**	0.19–0.46	**0.24**	0.13–0.43
	Chey Saen	0.97	0.48–1.77	**0.34**	0.20–0.58	**0.51**	0.25–0.97
	Choam Khsant	1.96	0.93–3.76	0.59	0.32–1.04	**2.33**	1.18–4.31
	Sangkom Thmei	0.9	0.39–1.96	**2.09**	1.13–4.04	**2.35**	1.04–4.70
	Kuleaen	1.33	0.62–2.51	1.1	0.61–1.98	**2.49**	1.25–5.09

^a^The relative rate ratio for each multinomial outcome category compares the risk to that of non-infected participants (baseline outcome group)

^b^Participants who never used the health facility

*Abbreviations: RRR* relative rate ratio (posterior median), *BCI* Bayesian credible interval

### Clustering tendency of *S. stercoralis* and hookworm mono- and co-infection

*Strongyloides stercoralis* exhibited almost no spatial correlation as indicated by the low location-specific variance and range, even in the absence of parameters. Hookworm mono-infection and co-infection exhibited moderate clustering tendencies, both with cluster sizes of about 20 km. Parameters of the three following multinomial geostatistical models are presented in Table 3: (i) model without covariates; (ii) predictive model including only environmental factors; and (iii) determinant analysis model including district and demographic, socioeconomic and behavioural factors.

**Table 3:** Model parameters of three geostatistical models

		Hookworm mono-infection		*S. stercoralis* mono-infection		Co-infection	
		Median	95% BCI	Median	95% BCI	Median	95% BCI
Model without covariates	σ^2a^	0.59	0.32–1.20	0.23	0.10–0.46	1.00	0.57–1.91
	Range (km)^b^	23.48	3.98–65.2	0.44	0.21–12.32	19.95	5.73–57.76
Predictive model^c^	σ^2a^	0.33	0.16–0.63	0.14	0.03–0.32	0.64	0.36–1.56
	Range (km)^b^	0.73	0.21–15.37	0.42	0.20–4.77	9.39	0.24–54.60
Determinant analysis model^d^	σ^2a^	0.14	0.03–0.32	0.22	0.08–0.49	0.33	0.18–0.60
	Range (km)^b^	0.41	0.20–4.27	0.72	0.21–19.7	0.46	0.20–7.36

^a^σ^2^ is the location-specific unexplained variance

^b^The range is the distance at which the spatial correlation becomes less than 5%

^c^Predictive model: geostatistical multinomial model used to predict *S. stercoralis* and hookworm mono- and co-infection risk at un-surveyed locations

^d^Determinant analysis model: model including only socio-demographic, behavioural and district identification data

### Prediction of *S. stercoralis* and hookworm mono- and co-infection risks

Of the three pairs (exchangeable vs geostatistical random effect) of models submitted to variable selection, model validation indicated that the geostatistical model, including LST day minimum, LST night mean, soil organic carbon, land use land cover, and RFE maximum, had the best predictive ability as assessed by the MSE. Results of the model validation are available in Additional file 3: Table S2. Relative risk ratios (RRR) of the predictive models are presented in Table 4.

**Table 4:** Results of the geostatistical multinomial predictive model. RRR in bold are significant at 5% level. Data were obtained from a cross-sectional survey conducted in 2010 in 60 villages of Preah Vihear province, North Cambodia, among 2576 participants aged over 1 year

		Hookworm mono-infection		*S. stercoralis* mono-infection		Co-infection	
		RRR^a^	95% BCI	RRR^a^	95% BCI	RRR^a^	95% BCI
LST day, year minimum		**0.73**	0.56–0.95	0.98	0.78–1.24	**0.60**	0.41–0.80
LST night, year mean		**0.79**	0.62–0.99	1.03	0.88–1.25	**0.66**	0.50–0.89
Rainfall, year maximum		1.27	0.99–1.60	**0.79**	0.66–0.95	0.91	0.67–1.62
Soil organic carbon (g/kg)	5.00–9.99			1.00			
	10.00–19.99	0.90	0.57–1.41	**0.57**	0.40–0.86	0.58	0.32–1.42
Land use/land cover	Savanna			1–00			
	Forests	1.02	0.52–2.00	1.59	0.92–2.78	0.87	0.42–1.95
	Grassland	0.79	0.30–2.07	1.52	0.77–3.04	2.13	0.73–5.84
	Cropland and crop-natural vegetation mosaic	1.18	0.64–2.02	**1.71**	1.12–2.61	**2.20**	1.22–3.79

^a^The relative rate ratio for each outcome category compares the risk of each infection group to that of non-infected participants (baseline outcome group)

*Abbreviations: RRR* relative rate ratio (posterior median), *BCI* Bayesian credible interval

**Fig. 1:**
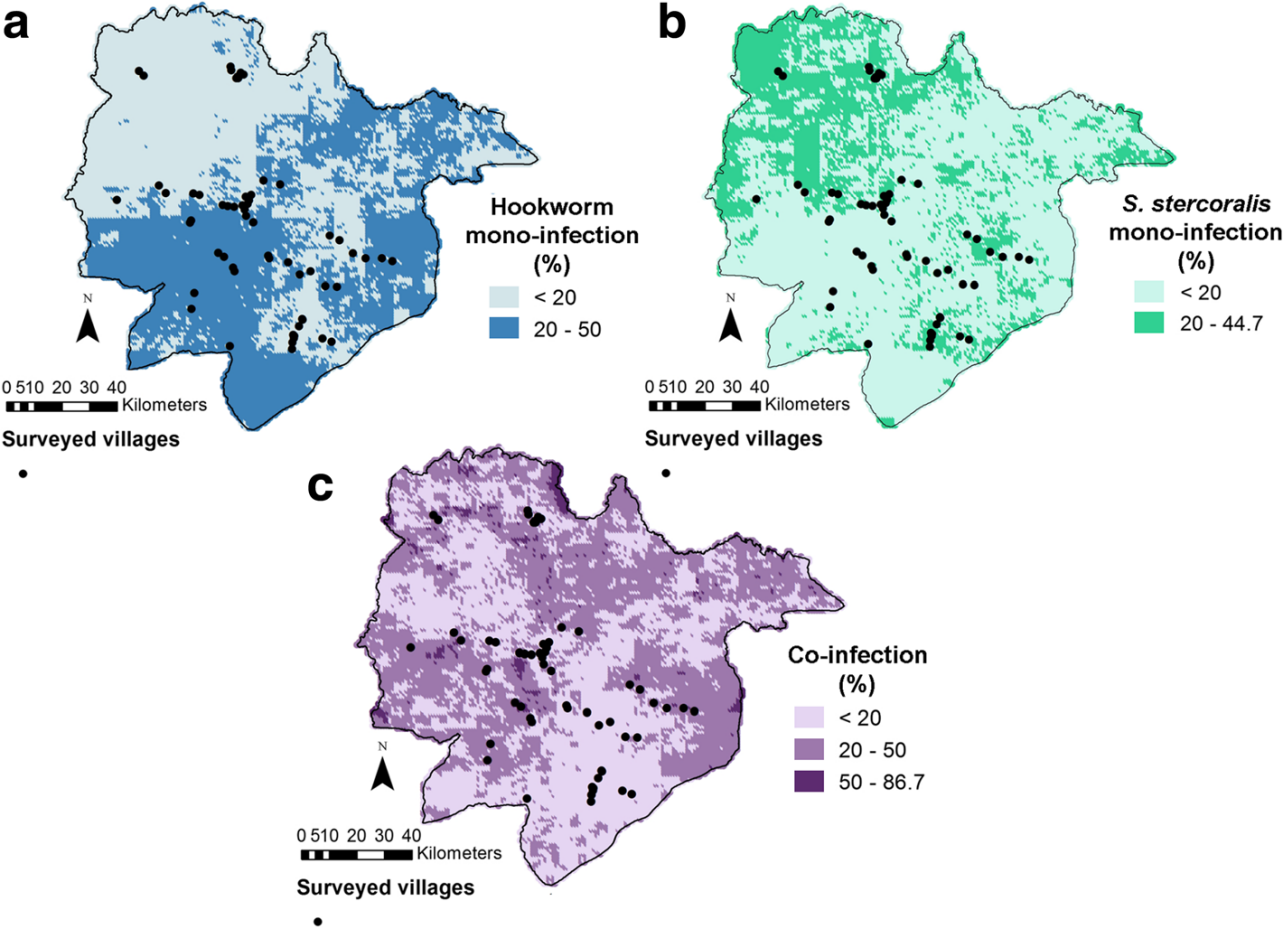
Maps of predicted hookworm mono-infection (**a**), *S. stercoralis* mono-infection (**b**) and co-infection (**c**) risk in Preah Vihear Province, North Cambodia. Predictions correspond to the posterior median obtained with the geostatistical multinomial model described in Table 3

**Fig. 2:**
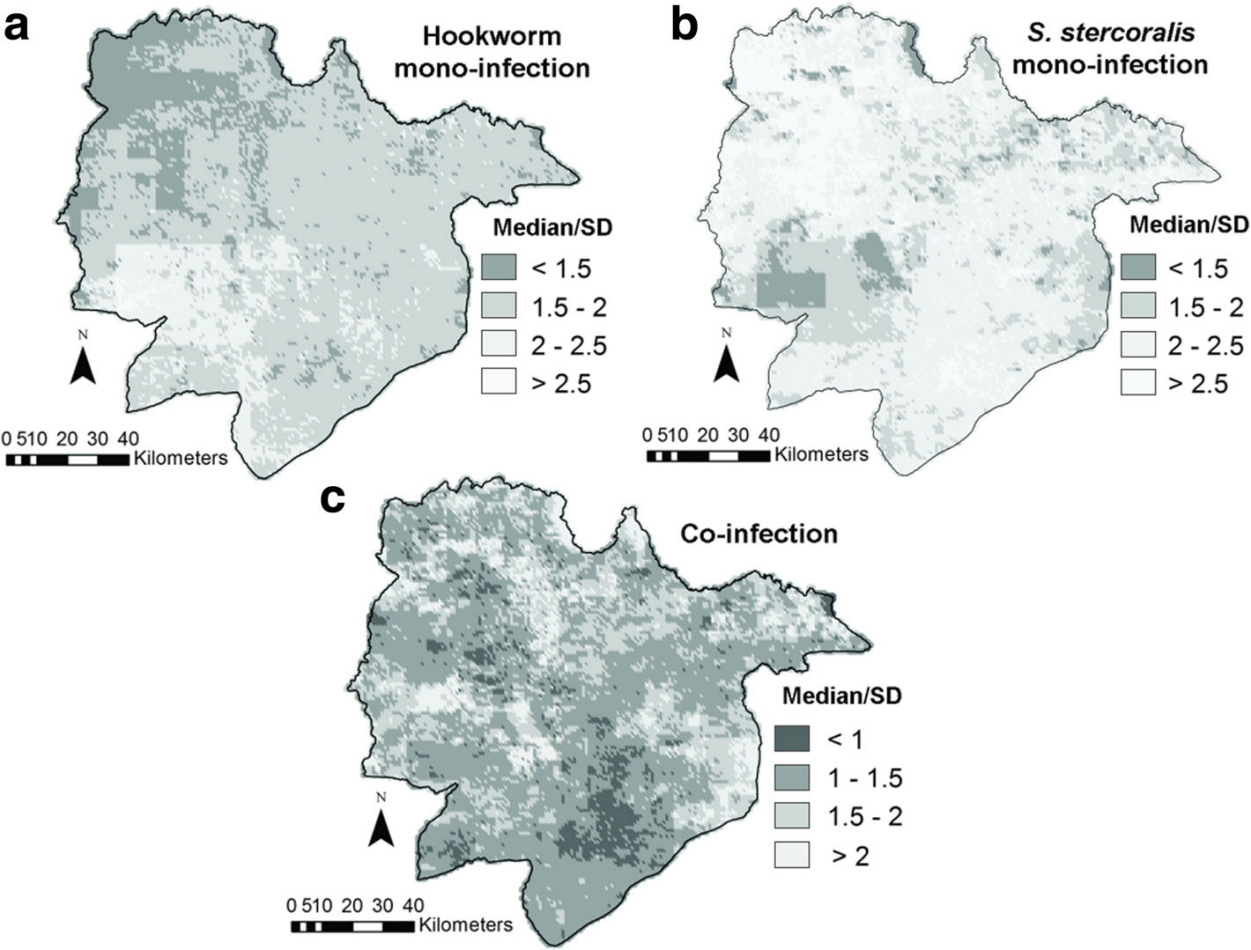
Error coefficients of the predicted hookworm mono-infection (**a**), *S. stercoralis* mono-infection (**b**) and co-infection (**c**) risk in Preah Vihear Province, North Cambodia. The error coefficient is the ratio between predicted median and its standard deviation. Darker zones indicate higher uncertainty

Figure 1 presents the maps of predicted *S. stercoralis* and hookworm mono- and co-infection risks. The two parasites, as well as co-infection cases, were ubiquitous in the province. The distributions of mono-infection with each parasite appeared complementary, with relatively higher risk of *S. stercoralis* and lower risk of hookworm in the North-West. The highest rates of co-infection were found in the South-West, East and North of the area. Model uncertainty for each predicted outcome, as expressed by the ratio of the posterior median over its standard deviation (error coefficient), is presented in Fig. 2. A smaller value indicates a larger degree of uncertainty.

## Discussion

This first *S. stercoralis-*hookworm co-risk map underlines the ubiquity of the two parasites and high prevalence rates in Preah Vihear Province, with more than two in three participants infected with one of the parasites and almost half (44%) experiencing co-infections.

In Cambodia, *S. stercoralis* infection was recently found to be associated with significant morbidity, and calls for control have been acknowledged [12, 42]. The hookworm situation, whereby half of the study participants experienced mostly light infections (2% both for moderate and heavy intensity classes), raises some questions. Infection levels were particularly high in school-aged children (SAC), with a prevalence rate similar to that of the late nineties, and infection intensity levels remaining above the 1% WHO control target for each intensity class [28, 29, 43–46].

STH control programmes reportedly achieve high coverage rates in Cambodia, but the assessment methods remain unclear, particularly with respect to the impact of school enrolment (net ratio was 65.2% in 2005 in Preah Vihear) [28, 29, 47]. An important aspect of programme evaluation is to estimate both coverage, i.e. the proportion of eligible population who received tablets, and compliance, i.e. the proportion of eligible population who actually swallowed tablets; these components can be substantially different, with the latter being the best measure of programme effectiveness [48].

Additionally, STH control programmes in Cambodia deliver mebendazole, which has a significantly weaker effect on hookworm infection than albendazole [49–51]. Decreased mebendazole efficacy against hookworm has raised concerns in Southeast Asia [51–53]. Reasons for its low performance should be investigated and explore both potential resistance and efficacy on *Ancylostoma ceylanicum*, a hookworm of dogs and cats that is highly prevalent among humans in Southeast Asia, and in Preah Vihear Province [54–58].

Another cause of persistent hookworm infection might be high reinfection rates, which can reach around 60% one year post-treatment for hookworm [23]. Documenting hookworm reinfection rates in the country might help disentangle the reasons why hookworm infection levels remain high [24].

Individuals living in the poorest households had higher odds of co-infection or mono-infection with any parasite, which reconfirms that STH infections, including *S. stercoralis*, are diseases of poverty [3, 7, 59]. The use of a robust method to construct the socioeconomic index might explain this result, since *S. stercoralis* was not associated with socioeconomic status in the few studies that accounted for it [13, 55]. The higher odds of infection with either parasite alone or concurrently among males were in line with results from other studies conducted in Asia [13, 55, 60–63]. School-aged children and adolescents had the highest risk of being co-infected, suggesting that this age group is an important target for control of both parasites in this setting. Treatment decreased the odds of hookworm mono-infection, but did not affect the odds of being infected with *S. stercoralis,* against which it is not effective at a single oral dose [49]. Defecating in toilets, boiling drinking water and having been treated with anthelmintics were protective factors against co-infection. The role of sanitation in STH transmission is widely acknowledged, whereas the association between systematically boiling drinking water and co-infection is unexpected, given the infection route of these two parasites [64–67]. This variable most likely acted as a proxy for overall hygienic health-promoting behaviour. Yet, an oral infection route has not been excluded for *S. stercoralis*; *Ancylostoma duodenale* can also infect orally, although it occurs rarely in this setting [57, 68, 69].

The spatial dependence of co-infection was mostly attributable to hookworm, as *S. stercoralis* exhibited almost no clustering tendency, even in the absence of covariates. This does not preclude, however, that *S. stercoralis* risk would cluster at a larger scale. Environmental factors partly explain the clustering of all outcomes (as indicated by the decrease in unexplained variance (σ^2^) and the drop of the range after inclusion of environmental variables). Climatic parameters influencing the distribution of the two parasites have been discussed elsewhere. Two previously identified factors are increased temperature and rainfall, either preventing or favouring larvae survival through desiccation or wetness, respectively [4, 12, 70]. Model parameters indicate that socio-demographic and behavioural variables played an important role in explaining the spatial variation of hookworm mono-infection and co-infection, but less so for *S. stercoralis* mono-infection. This result might reflect variability in mebendazole PC effectiveness across provincial regions, which cannot be excluded, despite adjusting for anthelmintic treatment. Indeed, we only adjusted for self-reported treatment, which is subject to recall bias, particularly after several deworming rounds [71]. We analysed data from 2010; since then, STH control with mebendazole has been scaled up to include additional age groups. Up-to-date studies accounting for programme coverage and compliance would help to assess the respective roles of environment, control and behaviour on infection levels with the two parasites.

Our study has some limitations. Although including only the negative participants with complete results ensured high specificity while keeping the maximum number of positive observations in the sample, it resulted in overestimated prevalence rates for both parasites, i.e. 48.6 vs 44.7% with a complete case analysis for *S. stercoralis* and 49.0 vs 39.6% for hookworm. Additionally, we did not address infection intensity of hookworm infection but most cases were of light intensity and geostatistical predictive models for infection intensity usually yield substantial uncertainty, particularly in low intensity settings [62, 72, 73]. Children under six years were under-represented in the sample, due to insufficient stool amounts for performing the Baermann technique, a shortcoming that will keep occurring as long as combining Baermann and KAP provides the most sensitive diagnostic approach for prevalence surveys. This aspect underlines the need for new techniques to diagnose *S. stercoralis* in endemic settings [74, 75]. No established prevalence thresholds exist for *S. stercoralis,* so we used those defined for other STH, although infection levels relating to the public health importance of this parasite might differ [10, 42]. The relationship between *S. stercoralis* infection intensity and morbidity, including potential co-morbidity arising from multiple infections needs to be investigated [76, 77].

There is a general agreement that *S. stercoralis,* now recognised as a major public health problem in Cambodia, needs to be integrated into existing STH control programmes [12, 13, 16, 24, 42, 78]. Under the present national policy, this integration would result in combining mebendazole with ivermectin, a combination found to be safe among schoolchildren in Zanzibar, but that has otherwise been rarely investigated [50, 79–81]. The combination of ivermectin with albendazole has largely proven to be safe for long-term use to control filarial diseases and also appears to add-value in the treatment of STH, including hookworm. [21, 46, 49, 79, 80, 82–87].

Drug combination is usually associated with a lower risk of emerging resistance, however the appearance of resistance to benzimidazoles might be accelerated by combining it with ivermectin, so any co-distribution should be closely monitored [88]. Cost-effectiveness studies of *S. stercoralis* control options, including the ancillary benefits of extending hookworm control coverage to adults and a potential switch to albendazole, are also needed.

The high impact that community-based, targeted ivermectin treatment achieved in Cambodia suggests that *S. stercoralis* control with PC is feasible. This assertion is supported by studies in several countries that documented a high ancillary impact of large-scale distribution of ivermectin against filarial diseases on *S. stercoralis* [42, 79, 89–91]. Still, longitudinal studies should be conducted to further confirm the impact of community-based PC with ivermectin in endemic settings of various transmission levels [24].

A major issue limiting the implementation of *S. stercoralis* control in Cambodia is the high cost of ivermectin, which is not donated in areas free of onchocerciasis. Ivermectin subsidization, donation, or production in the form of affordable generics are more likely once *S. stercoralis* is recognised as a public health problem and listed in the WHO PC strategy. A sensitive rapid diagnostic test would be crucial for efficient data collection and prevalence estimation at large scale [75].

In the meantime, the efficacy of both benzimidazoles against human and canine hookworm species occurring in the region need to be assessed. If a suboptimal efficacy of mebendazole was to be confirmed in the country, WHO should consider donating albendazole to Cambodia, whatever the control status of *S. stercoralis*. Indeed, the lost benefits of continuing to distribute an ineffective drug against one of the most prevalent STH in the country would be unfortunate, particularly in a country that benefits from a well-established and effective control network, was among the first countries to achieve the WHO STH control targets, and has eliminated lymphatic filariasis and trachoma as public health problems; in brief, a country that has a clear capacity to further tackle helminth infections [28, 29, 53, 57, 92, 93, 94].

## Conclusions

Both *S. stercoralis* and hookworm were highly prevalent in the province. Benzimidazoles delivered to control other STH are not effective against *S. stercoralis* infection and ivermectin should be integrated into STH control programmes to address strongyloidiasis, which is highly prevalent in Cambodia and is associated with significant morbidity. Infection levels of hookworm, despite several years of biannual school-based de-worming with mebendazole, were also high, even among school-aged children. The potential heavy co-morbidity due to overlapping hookworm and *S. stercoralis* infections should be investigated. Additionally, the effectiveness of the ongoing STH control programme should be assessed, particularly with regard to the efficacy of mebendazole against hookworm infection, including the zoonotic *A. ceylanicum*, which commonly infects humans in Cambodia. Control programmes should also be assessed in terms of the coverage and compliance achieved. Finally, the high cost of ivermectin, at 10–40 USD per treatment, is a major obstacle to the implementing *S. stercoralis* PC control in the country. Subsidies or ivermectin donations are needed to start tackling *S. stercoralis*, which is a public health problem in Cambodia.

## Additional files

Additional file 1: Formulation of the multinomial model. (DOCX 49 kb)

Additional file 2: Table S1. Results of the variable selection using bivariate multinomial regressions. Data were obtained from a cross-sectional survey conducted in 2010 in 60 villages of Preah Vihear Province, North Cambodia, among 2502 participants aged 2 years and above. (DOCX 26 kb)

Additional file 3: Table S2. Results of model validation for predictive models. (DOCX 12 kb)
