# Supplementary material for: Strongyloides stercoralis and hookworm co-infection: spatial distribution and determinants in Preah Vihear Province, Cambodia
Source: Parasit Vectors. 2018 Jan 12;11:33. doi: 10.1186/s13071-017-2604-8 (PMC5767026; doi:10.1186/s13071-017-2604-8)
Supplement: Supplementary file 1 — Formulation of the multinomial model. (DOCX 49 kb) [file 13071_2017_2604_MOESM1_ESM.docx]

**Additional file 1:** Formulation of the multinomial model

Analysis of hookworm and *S. stercoralis* mono- and co-infection: multinomial model

Let Y*_jk_* and n*_j_* be the number of infected and number of screened, and let us define the probability of hookworm mono-infection (k = 1), *S. stercoralis* mono-infection (k = 2), co-infection with the two species (k = 3) and of no infection ( k = 4) at each surveyed village j, j = 1,…. 60.

We assume that $Y_{jk}$follows a multinomial distribution, $Y_{jk} \sim MN(n_{j} , p_{jk})$ and model the log odds of infection in each multinomial category *h*, *h* = 1…3, *vs.* the baseline category (no infection) as follows:

${log(p}_{jh}/p_{j4})$ = α +$\sum_{l=1}^{n} \beta_{lh}X_{jhl}+\phi_{j}$, where $p_{jh}/p_{j4}$ is the risk ratio between infection status and no infection, *X_hlj_* is the i^th^ explanatory variable, $\beta_{lh}$ are the vector coefficients for each explanatory variable and multinomial category *h*, and $\phi_{j}$ is the locational random effect for each village (see below).

Random effects

We consider $\phi_{h}={({\phi h}_{1}, \ldots, \phi_{h51})}^{T}$ , where *h* = 1 in the logistic model and *h* = 1 … 3 in the multinomial model (each multinomial category has a specific random effect) , to be either an exchangeable random effect (non-spatial models) or a geostatistical random effect (spatial models). In particular, we assume *ϕ* ~ N(0 , σ^2^R*_ij_*) where σ^2^ is the variance parameter and R*_ij._*is the correlation matrix between locations. Under an exchangeable prior distribution R*_ij_* = 0 if i ≠ *j* and R*_ij_* = 1 if *i* = *j_._* Under the assumption of a spatial stationary isotropic process,$R_{ij}=exp(d_{ij}, \rho)$, where *d_ij_* is the Euclidean distance between two locations *s_i_* and *s_j_*, and *ρ* is a measure of how spatial correlation decreases with the distance. The distance at which the spatial correlation between villages gets under 5% is equal to 3/*ρ* and is called the range.

A vague inverse gamma prior with mean 1 and variance 100 was chosen for σ^2^, and a uniform prior for *ρ* with parameters calculated as a function of the minimum and maximum distance between sampled villages was adopted, that is:

$\rho\sim dunif(-\frac{\log(0.01)}{\min\text{distance}};-\frac{\log(0.01)}{\max\text{distance}})$.

We chose a vague Normal distribution with a mean of zero and a variance of 1000 for all the regression coefficients.
