# Supplementary material for: Strongyloides stercoralis and hookworm co-infection: spatial distribution and determinants in Preah Vihear Province, Cambodia
Source: Parasit Vectors. 2018 Jan 12;11:33. doi: 10.1186/s13071-017-2604-8 (PMC5767026; doi:10.1186/s13071-017-2604-8)
Supplement: Supplementary file 2 — Results of the variable selection using bivariate multinomial regressions. Data were obtained from a cross-sectional survey conducted in 2010 in 60 villages of Preah Vihear Province, North Cambodia, among 2502 participants aged 2 years and above. (DOCX 26 kb) [file 13071_2017_2604_MOESM2_ESM.docx]

**Additional file 2: Table S1.** Results of the variable selection using bivariate multinomial regressions**.** Data were obtained from a cross-sectional survey conducted in 2010 in 60 villages of Preah Vihear Province, North Cambodia, among 2,502 participants aged 2 years and above.

| **Variable** | **Category** | ***S. stercoralis*-hookworm co-infection** | | |  | **Hookworm mono-infection** | | |  | ***S. stercoralis* mono-infection** | | |
| --- | --- | --- | --- | --- | --- | --- | --- | --- | --- | --- | --- | --- |
|  |  | **OR** | **95% CI** | **LRT p-value** |  | **OR** | **95% CI** | **LRT p-value** |  | **OR** | **95% CI** | **LRT p-value** |
| Sex | Male | 1.00 |  |  |  |  |  |  |  |  |  |  |
|  | Female | 0.58 | 0.47 - 0.70 | < 0.0001 |  | 0.79 | 0.63 - 0.99 | < 0.0001 |  | 0.58 | 0.46 - 0.72 | < 0.0001 |
| Age (years) | 6-18 | 1.00 |  |  |  |  |  |  |  |  |  |  |
|  | < 6 | 0.37 | 0.26 - 0.55 | < 0.0001 |  | 0.33 | 0.2 - 0.55 | < 0.0001 |  | 0.52 | 0.33 - 0.83 | < 0.0001 |
|  | 19-49 | 0.85 | 0.68 - 1.06 |  |  | 1.17 | 0.91 - 1.49 |  |  | 1.38 | 1.07 - 1.79 |  |
|  | ≥ 50 | 1.03 | 0.74 - 1.44 |  |  | 0.99 | 0.67 - 1.46 |  |  | 1.88 | 1.31 - 2.70 |  |
| Occupation | Rice farmer | 1.00 |  |  |  |  |  |  |  |  |  |  |
|  | At home, other | 0.69 | 0.53 - 0.90 | < 0.0001 |  | 0.46 | 0.33 - 0.63 | < 0.0001 |  | 0.56 | 0.41 - 0.76 | < 0.0001 |
|  | School | 0.89 | 0.71 - 1.11 |  |  | 0.85 | 0.67 - 1.09 |  |  | 0.62 | 0.47 - 0.80 |  |
| Socioeconomic status | Least poor | 1.00 |  |  |  |  |  |  |  |  |  |  |
|  | Poor | 1.18 | 0.93 - 1.49 | < 0.0001 |  | 1.02 | 0.78 - 1.34 | < 0.0001 |  | 1.07 | 0.82 - 1.39 | < 0.0001 |
|  | Poorest | 1.80 | 1.41 - 2.30 |  |  | 1.94 | 1.48 - 2.55 |  |  | 1.26 | 0.95 - 1.67 |  |
| Level of education | Primary school | 1.00 |  |  |  |  |  |  |  |  |  |  |
|  | No school | 1.21 | 0.98 - 1.50 | 0.2188 |  | 0.92 | 0.72 - 1.18 | 0.2188 |  | 1.01 | 0.79 - 1.30 | 0.2188 |
|  | Secondary school or higher | 0.94 | 0.66 - 1.34 |  |  | 1.04 | 0.70 - 1.52 |  |  | 1.2 | 0.82 - 1.75 |  |
| Reported regular defecation place | Forest | 1.00 |  |  |  |  |  |  |  |  |  |  |
|  | Toilet | 0.24 | 0.17 - 0.36 | < 0.0001 |  | 0.41 | 0.28 - 0.60 | < 0.0001 |  | 0.61 | 0.43 - 0.85 | < 0.0001 |
|  | Rice field of water | 1.18 | 0.85 - 1.63 |  |  | 0.65 | 0.44 - 0.98 |  |  | 0.89 | 0.62 - 1.30 |  |
|  | Behind the house | 1.01 | 0.81 - 1.27 |  |  | 0.86 | 0.67 - 1.11 |  |  | 0.68 | 0.52 - 0.89 |  |
| Wearing shoes, frequency | Often or always | 1.00 |  |  |  |  |  |  |  |  |  |  |
|  | Sometimes or never | 0.74 | 0.57 - 0.96 | < 0.0001 |  | 0.55 | 0.40 - 0.76 | < 0.0001 |  | 0.37 | 0.26 - 0.54 | < 0.0001 |
| Washing hands after defecating | Yes | 1.00 |  |  |  |  |  |  |  |  |  |  |
|  | No or don't know | 1.13 | 0.91 - 1.41 | 0.5877 |  | 1.17 | 0.91 - 1.50 | 0.5877 |  | 1.07 | 0.83 - 1.38 | 0.5877 |
| Washing hands before eating | Yes | 1.00 |  |  |  |  |  |  |  |  |  |  |
|  | No or don't know | 1.18 | 0.83 - 1.69 | 0.7014 |  | 0.95 | 0.62 - 1.45 | 0.7014 |  | 1 | 0.66 - 1.52 | 0.7014 |
| Using soap or ashes when washing hands | No | 1.00 |  |  |  |  |  |  |  |  |  |  |
|  | Yes | 0.82 | 0.66 - 1.02 | 0.118 |  | 0.78 | 0.61 - 1.00 | 0.118 |  | 0.97 | 0.77 - 1.24 | 0.118 |
| Ever treated for worms | Yes | 1.00 |  |  |  |  |  |  |  |  |  |  |
|  | No or don't know | 0.50 | 0.40 - 0.62 | < 0.0001 |  | 0.58 | 0.45 - 0.74 | < 0.0001 |  | 0.68 | 0.54 - 0.86 | < 0.0001 |
| Do you know anything about worms? | No | 1.00 |  |  |  |  |  |  |  |  |  |  |
|  | Yes | 1.20 | 0.94 - 1.55 | 0.0875 |  | 1.11 | 0.83 - 1.48 | 0.0875 |  | 1.42 | 1.08 - 1.88 | 0.0875 |
| Ever used the health facility | Yes | 1.00 |  |  |  |  |  |  |  |  |  |  |
|  | No | 0.71 | 0.56 - 0.90 | 0.044 |  | 0.9 | 0.69 - 1.17 | 0.044 |  | 0.89 | 0.68 - 1.15 | 0.044 |
| Distance to health facility (minutes) | Close (1 to 20 minutes) | 1.00 |  |  |  |  |  |  |  |  |  |  |
|  | Less close (21 to 30 minutes) | 1.64 | 1.26 - 2.13 | 0.0004 |  | 1.36 | 1.01 - 1.83 | 0.0004 |  | 1.18 | 0.88 - 1.58 | 0.0004 |
|  | Least close (≥ 31 minutes) | 1.69 | 1.26 - 2.25 |  |  | 1.45 | 1.05 - 2.01 |  |  | 0.97 | 0.69 - 1.36 |  |
|  | Not applicable | 1.00 | 0.76 - 1.33 |  |  | 1.13 | 0.82 - 1.54 |  |  | 0.94 | 0.69 - 1.27 |  |
| Toilet at home | No | 1.00 |  |  |  |  |  |  |  |  |  |  |
|  | Yes | 0.24 | 0.17 - 0.35 | < 0.0001 |  | 0.44 | 0.31 - 0.63 | < 0.0001 |  | 0.73 | 0.53 - 0.996 | < 0.0001 |
| Main water source for general use | Open water body, rain |  |  |  |  |  |  |  |  |  |  |  |
|  | Well | 0.50 | 0.34 - 0.75 | 0.0133 |  | 0.56 | 0.36 - 0.88 | 0.0133 |  | 0.6 | 0.38 - 0.95 | 0.0133 |
|  | Wellpump | 0.50 | 0.33 - 0.76 |  |  | 0.66 | 0.41 - 1.06 |  |  | 0.69 | 0.43 - 1.12 |  |
| Source of drinking water, wet season | Well |  |  |  |  |  |  |  |  |  |  |  |
|  | Wellpump | 0.98 | 0.75 - 1.28 | 0.1194 |  | 1.24 | 0.93 - 1.66 | 0.1194 |  | 1.06 | 0.79 - 1.42 | 0.1194 |
|  | Rain | 1.12 | 0.88 - 1.42 |  |  | 1.24 | 0.94 - 1.62 |  |  | 0.99 | 0.75 - 1.30 |  |
|  | Open water body | 1.86 | 1.21 - 2.88 |  |  | 1.86 | 1.14 - 3.02 |  |  | 1.32 | 0.79 - 2.21 |  |
| Source of drinking water, dry season | Well |  |  |  |  |  |  |  |  |  |  |  |
|  | Wellpump | 0.98 | 0.79 - 1.21 | 0.036 |  | 1.13 | 0.89 - 1.44 | 0.036 |  | 1.22 | 0.96 - 1.55 | 0.036 |
|  | Open water body, rain | 1.78 | 1.18 - 2.68 |  |  | 1.55 | 0.97 - 2.48 |  |  | 1.26 | 0.76 - 2.08 |  |
| Boiling drinking water | Never |  |  |  |  |  |  |  |  |  |  |  |
|  | Yes during dry or wet season but not both | 1.22 | 0.82 - 1.81 | 0.0001 |  | 1.79 | 1.18 - 2.69 | 0.0001 |  | 1.03 | 0.64 - 1.66 | 0.0001 |
|  | Yes both dry and wet season | 0.64 | 0.50 - 0.83 |  |  | 0.79 | 0.60 - 1.05 |  |  | 1.09 | 0.83 - 1.42 |  |
| Own dog | No |  |  |  |  |  |  |  |  |  |  |  |
|  | Yes | 0.77 | 0.63 - 0.95 | 0.0064 |  | 0.9 | 0.71 - 1.13 | 0.0064 |  | 1.16 | 0.91 - 1.48 | 0.0064 |
| Own farm animals | No |  |  |  |  |  |  |  |  |  |  |  |
|  | Yes | 0.61 | 0.43 - 0.88 | 0.066 |  | 0.72 | 0.48 - 1.10 | 0.066 |  | 0.74 | 0.49 - 1.14 | 0.066 |
| Number of family members |  | 1.01 | 0.96 - 1.06 | 0.3535 |  | 0.96 | 0.91 - 1.02 | 0.3535 |  | 1.01 | 0.96 - 1.07 | 0.3535 |
| Land use/Land cover | Savanna |  |  |  |  |  |  |  |  |  |  |  |
|  | Forests | 1.09 | 0.77 - 1.55 | < 0.0001 |  | 0.94 | 0.65 - 1.37 | < 0.0001 |  | 1.46 | 0.98 - 2.18 | < 0.0001 |
|  | Grassland | 0.53 | 0.35 - 0.80 |  |  | 0.38 | 0.23 - 0.61 |  |  | 1.15 | 0.75 - 1.75 |  |
|  | Cropland and crop-natural vegetation mosaic | 1.15 | 0.92 - 1.45 |  |  | 0.67 | 0.52 - 0.86 |  |  | 1.52 | 1.16 - 2.00 |  |
| Soil organic carbon (g/kg) | 5.00 – 9.99 |  |  |  |  |  |  |  |  |  |  |  |
|  | 10.00 – 19.99 | 0.37 | 0.30 - 0.45 | < 0.0001 |  | 0.51 | 0.41 - 0.64 | < 0.0001 |  | 0.69 | 0.55 - 0.86 | < 0.0001 |
| LST day, year minimum |  | 0.80 | 0.74 - 0.86 | < 0.0001 |  | 0.77 | 0.71 - 0.83 | < 0.0001 |  | 1.02 | 0.94 - 1.11 | < 0.0001 |
| LST night, year mean |  | 0.35 | 0.28 - 0.44 | < 0.0001 |  | 0.47 | 0.36 - 0.60 | < 0.0001 |  | 0.98 | 0.75 - 1.26 | < 0.0001 |
| Rainfall, year maximum |  | 1.09 | 1.01 - 1.17 | < 0.0001 |  | 1.3 | 1.19 - 1.41 | < 0.0001 |  | 0.91 | 0.83 - 0.99 | < 0.0001 |
| District | Tbaeng Mean Chey |  |  |  |  |  |  |  |  |  |  |  |
|  | Rovieng | 0.33 | 0.24 - 0.45 | < 0.0001 |  | 0.3 | 0.22 - 0.41 | < 0.0001 |  | 0.77 | 0.56 - 1.07 | < 0.0001 |
|  | Chey Saen | 0.75 | 0.53 - 1.07 |  |  | 0.41 | 0.28 - 0.60 |  |  | 1.14 | 0.79 - 1.65 |  |
|  | Choam Khsant | 1.98 | 1.42 - 2.75 |  |  | 0.51 | 0.34 - 0.77 |  |  | 1.62 | 1.11 - 2.36 |  |
|  | Sangkom Thmei | 2.79 | 1.83 - 4.25 |  |  | 2.05 | 1.33 - 3.16 |  |  | 0.99 | 0.56 - 1.74 |  |
|  | Kuleaen | 2.28 | 1.59 - 3.26 |  |  | 1.01 | 0.67 - 1.50 |  |  | 1.28 | 0.82 - 1.99 |  |

*Abbreviations*: OR, odds ratio; CI, confidence interval; LRT, likelihood ratio test.

^a^ The relative rate ratio for each outcome category compares the risk to that of non-infected participants (baseline outcome group).

^b^ open water: pond canal river lake dam.
