# Supplementary material for: Strongyloides stercoralis and hookworm co-infection: spatial distribution and determinants in Preah Vihear Province, Cambodia
Source: Parasit Vectors. 2018 Jan 12;11:33. doi: 10.1186/s13071-017-2604-8 (PMC5767026; doi:10.1186/s13071-017-2604-8)
Supplement: Supplementary file 3 — Results of model validation for predictive models. (DOCX 12 kb) [file 13071_2017_2604_MOESM3_ESM.docx]

**Additional file 3: Table S2.** Results of model validation for predictive models

| **Model** | **Total MSE** |
| --- | --- |
| Model 1, non-spatial | 508.31 |
| Model 1, spatial | 468.85 |
| Model 2, non-spatial | 722.94 |
| Model 2, spatial | 686.73 |
| Model 3, non-spatial | 682.84 |
| Model 3, spatial | 700.96 |

Model 1: LST Day minimum, LST night mean, Soil organic carbon, land use land cover, RFE maximum

Model 2: LST Day minimum, LST night mean, Soil organic carbon, land use land cover, district

Model 3: LST Day minimum, LST night mean, Soil organic carbon, land use land cover, RFE maximum, district

non-spatial: including an exchangeable random effect

spatial: including a geostatistical random effect
